# Factors Influencing Stroke Severity Based on Collateral Circulation, Clinical Markers and Machine Learning

**DOI:** 10.3390/diagnostics15232983

**Published:** 2025-11-24

**Authors:** Jia-Lang Xu

**Affiliations:** Department of Applied Statistics, National Taichung University of Science and Technology, Taichung 404336, Taiwan; jlxu@nutc.edu.tw

**Keywords:** stroke prognosis, lateral branch circulation, random forest, XGBoost, Clinical Decision Support Systems

## Abstract

**Background/Objectives:** Stroke is a serious neurological disorder that significantly affects patients’ quality of life and overall health. The severity of a stroke can vary widely and is influenced by multiple factors, such as clinical presentation, diagnostic findings, and the site of onset. This study aimed to identify and analyze key variables that contribute to stroke severity, with a particular focus on the role of collateral circulation. **Methods**: This study analyzed clinical, imaging, and biochemical variables—ipsilateral collateral flow on MRA, MRI unilateral–bilateral stroke, systolic blood pressure (SBP), fasting plasma glucose (FPG), and blood urea nitrogen (BUN). Group differences used chi-square and Mann–Whitney U tests. Class imbalance was addressed with SMOTE; Logistic Regression, Random Forest, XGBoost, and SVM were cross-validated, reporting accuracy, precision, recall, and F1 with 95% CIs. **Results**: Reduced or absent ipsilateral collateral flow and unilateral–bilateral stroke were strongly associated with greater severity (*p* < 0.001). SBP was significant (*p* = 0.034), FPG was significant (*p* = 0.023), and BUN was borderline (*p* = 0.059). SMOTE improved prediction: Random Forest achieved accuracy 83.3% (CI: 79.1–87.6) and F1 84.0% (CI: 79.1–88.9); XGBoost reached accuracy 80.2% (CI: 71.5–89.0) and F1 81.4% (CI: 73.8–89.0). Logistic Regression improved to F1 70.8% (CI: 55.4–86.2), whereas SVM declined to accuracy 52.2% (CI: 37.5–67.0). **Conclusions**: Collateral status and unilateral–bilateral stroke are key determinants of severity; SBP and FPG add prognostic value, with BUN borderline. Tree-based ensembles trained on SMOTE-balanced data provide the most reliable predictions for risk stratification. These findings suggest that future work may focus on integrating such predictive models into Clinical Decision Support Systems (CDSSs) to enhance early risk identification, strengthen CDSSs, and enable more personalized care planning for stroke patients.

## 1. Introduction

Stroke is the second leading cause of death and the third leading cause of disability worldwide [1]. In Taiwan, it remains the third leading cause of death, with a worrisome trend toward younger onset [2]. In clinical practice, patients with acute stroke are usually stabilized in the emergency department before referral to neurology or neurosurgery for further management. Prior to admission, vascular status is commonly assessed through carotid duplex sonography (CDS) and magnetic resonance angiography (MRA). CDS provides information on carotid atherosclerosis, including stenosis or occlusion of the extracranial arteries [3], while MRA offers a comprehensive view of intracranial and extracranial vessels, enabling detection of arterial narrowing, occlusion, and ischemic tissue changes [4]. Collateral circulation plays a critical role in preserving cerebral perfusion in the presence of arterial occlusion. While it is less likely to be recruited when major cerebral arteries remain patent, patients with severe internal carotid artery (ICA) stenosis or occlusion frequently exhibit collateral pathways, such as those via the circle of Willis, which redirect blood flow to ischemic regions [5]. The adequacy of collateral circulation has been strongly linked to stroke severity and prognosis. For example, left-sided strokes are often associated with worse outcomes, and injury to eloquent brain regions such as the postcentral gyrus, nucleus accumbens, and insular cortex can further aggravate functional impairment [6, 7]. Several clinical and imaging markers have been identified as important predictors of stroke severity, including the contralateral common carotid artery (CCA) pulsatility index (PI), ipsilateral external carotid artery (ECA) resistance index (RI), hypoperfusion intensity ratio (HIR), Alberta Stroke Program Early CT Score (ASPECTS), and ipsilateral ECA PI [8]. In the field of stroke, time has emerged as a critical determinant, reflecting the interdependence of perfusion, tissue viability, and therapeutic opportunity in the management of acute ischemic stroke [9]. Ischemic stroke remains a leading cause of death and long-term disability worldwide. Even when patients survive the acute event, they often experience residual sequelae of varying severity, imposing substantial physical, psychological, and socioeconomic burdens on both patients and caregivers, while markedly diminishing quality of life. Ischemic stroke occurs when cerebral arteries are obstructed or narrowed, commonly due to atherosclerosis from cholesterol deposition or, in some cases, as a late effect of head and neck radiation therapy. The resulting reduction in cerebral blood flow deprives brain tissue of oxygen, leading to ischemic injury. Among all stroke subtypes, ischemic stroke accounts for the majority of cases [10]. Pathophysiologically, ischemic stroke is characterized by distinct features of ferroptosis, including lipid peroxidation and iron accumulation, which appear to be closely correlated and may serve as critical mechanisms of neuronal injury [11]. Cerebral ischemia also triggers a cascade of interconnected processes such as excitotoxicity, oxidative stress, inflammation, and programmed cell death [12]. Importantly, collateral circulation has been shown to provide substantial therapeutic benefits for patients with ischemic stroke [13]. In particular, among those undergoing mechanical thrombectomy, the presence of robust collateral flow is associated with more favorable prognostic outcomes, including improved functional status at discharge [14, 15]. One study argues that leptomeningeal collateral circulation (LMC) can effectively mitigate acute ischemic stroke (AIS) [16].

With the rapid advancement of artificial intelligence and its growing integration into Clinical Decision Support Systems (CDSSs) [17], AI-assisted diagnostic tools have increasingly been adopted across various clinical settings. Numerous studies have demonstrated that AI can support physicians in clinical decision-making and improve workflow efficiency. For example, in critical care, researchers have applied edge computing and AI techniques to facilitate ventilator weaning decisions, achieving promising results [18, 19]. In the nursing domain, logistic regression and random forest models have been utilized for variable selection and model construction to support fall-risk assessment [20, 21]. In hepatology, image segmentation techniques have been employed to assist in the evaluation of liver fibrosis [22]. Within the field of ischemic stroke, AI has been explored for multiple applications, including automated infarct segmentation, vessel occlusion detection, prognosis prediction, and risk stratification [23]. Furthermore, studies that integrate clinical and imaging data collected within the first 24 h of hospital admission have shown that AI can enhance clinical scoring systems and improve communication between healthcare providers and patients [24]. These diverse and impactful findings collectively highlight the transformative potential of AI in healthcare. This study seeks to contribute to the development of practical, efficient, and clinically applicable AI tools.

This study seeks to advance current understanding by evaluating the prognostic significance of collateral circulation in stroke outcomes, extending beyond the traditionally examined baseline characteristics, hemodynamic parameters, and functional measures. Our objectives are to identify risk factors associated with poor prognosis and to elucidate how the presence or absence of collateral flow influences long-term outcomes. Prior evidence demonstrates that the NIHSS is significantly associated with both ischemic and hemorrhagic stroke severity, particularly when scores exceed 15 compared to intermediate ranges of 5–15. Moreover, higher NIHSS scores have been linked to prolonged hospitalization and substantially greater medical costs [25]. By combining statistical analyses with machine learning approaches, this study aims to enhance the accuracy of risk prediction and contribute to the development of more effective secondary prevention strategies, thereby supporting individualized stroke care.

In addition, this study makes several noteworthy contributions:

• Confirmed that unilateral–bilateral stroke classification is a critical prognostic determinant, providing new evidence on the importance of lesion laterality in outcome prediction.
• Demonstrated that insufficient or absent collateral circulation is strongly associated with greater stroke severity, emphasizing the clinical relevance of collateral status in acute stroke evaluation.
• Showed that under small and imbalanced sample conditions, SMOTE combined with tree-based models yields the most robust and reliable predictive performance, offering practical methodological guidance for modeling in limited clinical datasets.

## 2. Materials and Methods

**Figure 1:**
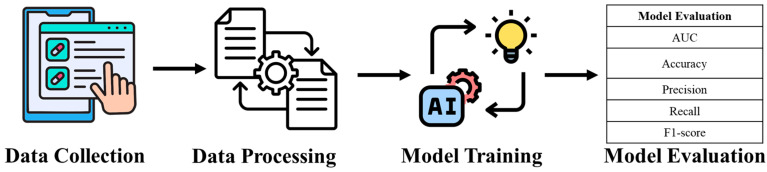
Workflow of the Proposed Clinical Data–Driven AI Prediction Framework.

Figure 1 presents an overview of the methodological workflow used in this study. The process begins with the collection and organization of clinical data via hospital information and documentation systems. These data are then processed through the proposed AI-based analytical pipeline, in which machine learning models perform feature extraction, pattern learning, and predictive analysis. The final stage generates interpretable clinical insights that can assist clinicians in risk assessment, support decision-making, and enhance diagnostic efficiency. This figure highlights the end-to-end progression from raw clinical input to AI-driven predictive output, providing a clear visualization of the study’s methodological framework.

### 2.1. Ethical Approval

The study was approved by the Institutional Review Board of Changhua Christian Hospital (approval no.: 210806, date: 13 September 2021). The Institutional Review Board waived the need for informed consent, considering the retrospective nature of the data collected. All methods were performed in accordance with the relevant guidelines and regulations or the Declaration of Helsinki.

### 2.2. Study Setting, Design, and Ethical Considerations

**Figure 2:**
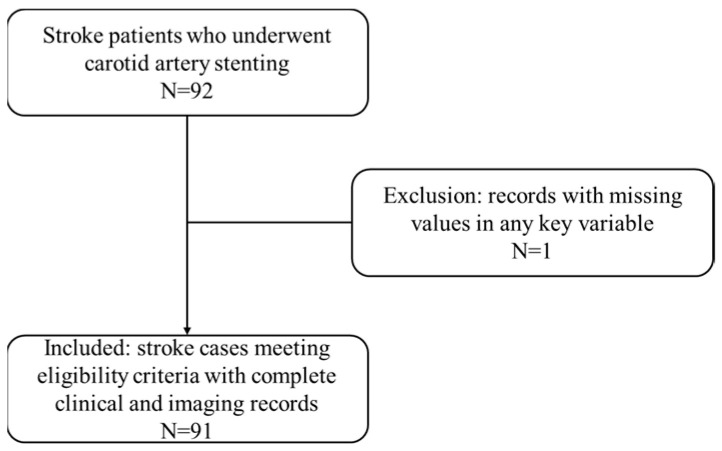
Study flow diagram for the carotid artery stenting cohort.

This retrospective study analyzed medical records of consecutive patients who underwent bridging therapy—comprising intravenous thrombolysis (IVT) followed by intra-arterial thrombectomy (IAT) in conjunction with computed tomography angiography (CTA) and/or perfusion (CTP) imaging, between January 2012 and December 2020 at the angiography unit of the Department of Neuroimaging in a large medical center in Taiwan. Eligible patients were those aged 20 to 89 years who presented with middle cerebral artery (MCA) stenosis or near occlusion segments M1, M2, or M3 during the index ischemic stroke and completed a minimum of 12 months of follow-up after the thrombectomy procedure. Patients were excluded if they had intracerebral hemorrhage, cerebral arteriovenous malformations or aneurysms, experienced recurrent stroke during the follow-up period, were lost to follow-up, or did not complete the 12-month follow-up. As shown in Figure 2, we initially identified 92 stroke patients who underwent carotid artery stenting. One record with missing values in a key variable was excluded, yielding a final analytic sample of 91 patients who met eligibility criteria with complete clinical and imaging records. All imaging assessments, including the evaluation and grading of collateral circulation, were performed by board-certified neurologists specializing in neurovascular disease. Two readers independently reviewed the CTA/CTP images using a standardized grading system, and discrepancies were resolved by consensus.

### 2.3. Experimental Environment

In this study, data preprocessing and descriptive statistical analyses were first conducted using R. Subsequently, the Synthetic Minority Over-sampling Technique (SMOTE) was applied in Python 3.10 to address class imbalance, followed by the implementation of machine learning algorithms and model development using Python.

### 2.4. Statistical Analysis

Recent studies have applied the chi-square test to investigate the association between calcified plaque types and ipsilateral acute ischemic anterior circulation [26]. In addition, the chi-square test has also been utilized for feature identification and extraction in related analyses [27]. The Mann–Whitney U test has been utilized to identify factors associated with stroke risk [28]. In this study, we adopted a statistics-driven feature selection procedure: categorical variables were evaluated with chi-square and continuous variables with Mann–Whitney U, and variables meeting two-sided *p* < 0.05 were retained as input features for modeling.

### 2.5. Model Training

This study implemented four supervised classifiers in scikit learn to identify the most suitable model for prediction: Logistic Regression [29], Random Forest [30, 31], XGBoost [32] using its scikit learn interface, and Support Vector Machine [33]. To mitigate class imbalance, we applied the Synthetic Minority Oversampling Technique SMOTE [34] to generate additional minority class samples and obtain a more balanced training set. Models were trained and tuned both with SMOTE and without SMOTE to quantify the impact of data augmentation on performance. Logistic Regression served as a baseline linear model for comparison. Random Forest and XGBoost were selected as representative tree-based ensemble methods due to their robustness to nonlinear relationships, ability to handle mixed data types, and strong performance in structured clinical datasets. The SVM classifier was implemented using scikit-learn’s SVC function, corresponding to a C-SVM with a radial basis function (RBF) kernel. All models were trained and evaluated within a 5-fold cross-validation framework, with Synthetic Minority Oversampling Technique (SMOTE) applied to the training folds to address class imbalance. Model performance metrics included AUC, accuracy, precision, recall, and F1-score, each reported with corresponding 95% confidence intervals.

## 3. Results

### Descriptive Statistic

The NIHSS score is a well-established predictor of stroke outcomes, with scores ≥ 16 indicating a high risk of death or severe disability, and scores ≤ 6 generally associated with favorable recovery. Among the TOAST stroke subtypes, only lacunar infarctions have been shown to predict outcomes independently of NIHSS scores, likely due to their distinct pathophysiology and the relative preservation of cortical function. [35] In our study, the absence of collateral circulation was observed in 72% (*n* = 25) of patients with poor stroke prognosis (NIHSS > 16), suggesting a potential association between poor collateral status and unfavorable outcomes. This finding highlights the additive prognostic value of collateral circulation, beyond the NIHSS score alone, in predicting functional recovery after stroke.

Table 1 presents the comparison of both categorical and continuous variables between patients with poor and good stroke prognosis. For categorical variables, distributions were summarized as counts and percentages. Chi-square tests were used to evaluate group differences. Variables demonstrating significant associations (*p* < 0.10) included contralateral middle cerebral artery stenosis (MCAS, *p* = 0.004), unilateral–bilateral stroke (*p* = 0.024), and ipsilateral collateral circulation on MRA (*p* = 0.021). These results suggest that vascular and anatomical factors are strongly associated with post-stroke outcomes. For continuous variables, values were summarized as mean, minimum, and maximum, and differences between groups were examined using the Mann–Whitney U test. Several parameters reached statistical or borderline significance (*p* < 0.10), including systolic blood pressure (*p* = 0.050), blood urea nitrogen (*p* = 0.070), fasting plasma glucose (*p* = 0.077), contralateral common carotid artery resistance index (*p* = 0.092), ipsilateral internal carotid artery resistance index (*p* = 0.010), contralateral ICA RI (*p* = 0.086), ipsilateral plaque index (*p* = 0.012), and contralateral plaque index (*p* = 0.061). Among these, the ipsilateral plaque index exhibited the strongest correlation with prognosis, underscoring its potential utility as an indicator of unfavorable clinical outcomes.

Guided by the above table, we first selected the variables with the strongest evidence of association (*p* < 0.05) as input features for modeling. In the SMOTE-balanced dataset, these were unilateral–bilateral stroke (*p* < 0.001), ipsilateral collateral flow (MRA; *p* < 0.001), systolic blood pressure (*p* = 0.034), fasting plasma glucose (*p* = 0.023), contralateral CCA resistance index (*p* = 0.001), ipsilateral ICA resistance index (*p* = 0.007), and ipsilateral plaque index (*p* = 0.002). Variables with borderline evidence—BUN (*p* = 0.059) and contralateral plaque index (*p* = 0.066)—were reserved for sensitivity analyses, whereas contralateral MCA stenosis (CTA; *p* = 0.132) and contralateral ICA resistance index (*p* = 0.297) were excluded from the primary feature set. Consistent across data settings, unilateral–bilateral stroke, ipsilateral collateral flow (MRA), ipsilateral ICA resistance index, and ipsilateral plaque index remained significant; SBP, FPG, and contralateral CCA resistance index reached significance only after SMOTE, while contralateral MCA stenosis (CTA) was significant only before balancing and contralateral ICA resistance index shifted from borderline to non-significant as shown in Table 2.

Table 3 presents the area under the ROC curve (AUC) for all four models, along with the corresponding 95% confidence intervals. Logistic Regression achieved the highest AUC at 0.682 (CI 0.605–0.759), demonstrating moderate discriminative ability. Random Forest showed a comparable AUC of 0.682 (CI 0.502–0.862), although with wider confidence intervals reflecting variability across folds. XGBoost achieved an AUC of 0.575 (CI 0.412–0.738), indicating lower discriminative performance. The SVM model produced the weakest result, with an AUC of 0.432 (CI 0.302–0.561), suggesting limited capability in distinguishing between severity categories. These AUC findings further support the observation that class imbalance affected model performance under the non-SMOTE condition. Although the accuracy values appeared moderate, such as 72.5% for SVM and 69.1% for Logistic Regression, their ability to detect minority cases was poor. For example, Logistic Regression exhibited a recall of only 24.0%. The SVM model generated NA values for Precision, Recall, and F1-score because it predicted all samples as belonging to the majority class. This resulted in a confusion matrix with zero true positives for the minority class, leading to division-by-zero situations in these metrics. Such behavior is common for kernel-based SVMs trained on highly imbalanced datasets, where the decision boundary becomes dominated by the majority class. The relatively low AUC values for XGBoost and SVM confirm that these models had difficulty establishing effective separation between severe and non-severe cases. This reinforces the necessity of applying class-balancing techniques and supports the use of SMOTE in subsequent analyses.

Table 4 presents the AUC values and corresponding 95% confidence intervals for each classifier following the application of SMOTE. Random Forest achieved the highest discriminative performance, with an AUC of 0.940 (CI 0.900–0.980), indicating excellent ability to distinguish between severe and non-severe stroke cases. XGBoost similarly demonstrated strong discriminative capability, yielding an AUC of 0.907 (CI 0.847–0.966). Logistic Regression showed moderate performance with an AUC of 0.747 (CI 0.588–0.905), while SVM exhibited poor discrimination, with an AUC of 0.398 (CI 0.239–0.558), reflecting substantial difficulty in ranking cases according to severity. These AUC results are consistent with the classification performance metrics reported in Table 4. After applying SMOTE, Random Forest attained the strongest overall performance, with an accuracy of 83.3%, precision of 79.6%, recall of 89.4%, and an F1-score of 84.0%, demonstrating a strong balance between sensitivity and precision. XGBoost followed with an accuracy of 80.2%, precision of 78.3%, recall of 86.6%, and an F1-score of 81.4%, indicating excellent generalizability. Logistic Regression also showed improved stability after oversampling, reaching an F1-score of 70.8% and accuracy of 71.3%. In contrast, SVM performance declined notably after SMOTE, with accuracy falling to 52.2% and an F1-score of 51.7%. The RBF-kernel SVM is known to be sensitive to synthetically interpolated samples, and in small or imbalanced datasets, SMOTE may introduce minority instances that do not align well with the kernel’s distance-based structure. This distortion can lead to suboptimal decision boundaries and degraded performance. Such effects are less prominent in tree-based models, which explains the strong performance of Random Forest and XGBoost in this study.

## 4. Discussion

Previous studies on stroke outcome prediction have primarily emphasized patient demographics, blood biomarkers, and comorbidities, with relatively limited attention to imaging-derived quantitative features [36]. In contrast, the present study incorporated eleven variables spanning clinical, biochemical, and imaging domains, enabling a more comprehensive evaluation of prognostic determinants. Among these variables, ipsilateral collateral circulation (ICC), unilateral–bilateral stroke classification, systolic blood pressure (SBP), and blood urea nitrogen (BUN) emerged as the most consistently influential predictors across models. These findings highlight the importance of integrating diverse clinical parameters, particularly collateral flow and lesion laterality, when assessing stroke severity. To address the substantial class imbalance inherent in the dataset, SMOTE was applied during model development. Training on SMOTE-processed data significantly improved predictive performance across models, consistent with evidence that SMOTE enhances minority-class representation and overall classification accuracy in machine learning applications. This reflects a broader methodological distinction between machine learning and traditional statistical approaches: while classical statistical models focus on inference, hypothesis testing, and causal interpretation, machine learning excels in predictive performance and in modeling complex nonlinear relationships [37]. Its scalability and flexibility make it particularly suited for diagnostic and risk stratification tasks in clinical settings [38]. In this study, Random Forest achieved the strongest performance, with an accuracy of 83.3% and a 95% confidence interval of 79.1–87.6, outperforming Logistic Regression, XGBoost, and SVM. Before applying SMOTE, severe class imbalance led the SVM classifier to predict nearly all cases as the majority class. Consequently, the confusion matrix contained no true positives for the minority class, making Precision, Recall, and F1-score impossible to compute due to division by zero. This phenomenon is characteristic of kernel-based SVMs under extreme imbalance, where decision boundaries are dominated by the majority class. SMOTE was therefore essential to enable meaningful evaluation of minority-class performance, even though SVM accuracy declined after oversampling due to the sensitivity of RBF kernels to synthetic samples. Given the small sample size and imbalanced data distribution, several methodological safeguards were implemented to minimize overfitting. 5-fold cross-validation ensured that each model was repeatedly evaluated on unseen data, providing a rigorous estimate of generalization performance and reducing the likelihood that the models learned patterns specific to individual folds. Tree-based ensemble methods demonstrated additional resilience against overfitting through mechanisms such as random feature subsampling and bootstrapped aggregation, which reduce dependence on any single variable or training instance. Across all models, the observed differences between training and validation performance remained within acceptable bounds, indicating that the predictive patterns captured were meaningful rather than artifacts of the training data. Collectively, these results demonstrate that the analytical framework adopted in this study effectively balanced predictive accuracy, generalization, and overfitting control, producing robust and clinically relevant findings that advance the understanding of prognostic factors in stroke severity.

Collateral circulation serves as a vital compensatory mechanism during arterial stenosis or occlusion, redirecting blood flow through alternative pathways to maintain cerebral perfusion and reduce tissue damage [5]. Patients with internal carotid artery (ICA) stenosis greater than 75% or complete occlusion exhibit a markedly higher likelihood of developing ipsilateral collateral circulation (ICC). While typically latent under normal conditions, this mechanism becomes functionally important in the presence of vascular obstruction. Conversely, in cases of severe ICA stenosis without adequate collateral support, the brain is left vulnerable to hypoperfusion, increasing both the risk and severity of ischemic stroke [39]. Moreover, isochronous collateral flow has been linked to recurrent ipsilateral ischemic events in patients with symptomatic carotid stenosis [40] and to greater hemodynamic compromise and ischemic burden [6]. Prognosis is also strongly influenced by unilateral–bilateral stroke and the extent of structural brain injury. Lesions within the left hemisphere are often associated with more severe neurological deficits and worse outcomes. In this study, MRI findings demonstrated that patients with a modified baseline MBD NIHSS score greater than 16 were distributed as follows: six cases with 0–50% stenosis, eight cases with 50–75% stenosis, and eleven cases with 75–99% stenosis. Damage to regions such as the amygdala and caudate nucleus has been associated with poor prognosis [41], while injury to the white matter, postcentral gyrus, nucleus accumbens, and insula correlates with impaired functional recovery. Notably, lesions affecting the left postcentral gyrus and inferior parietal lobule have been significantly linked to the severity of apraxia one year post-stroke [7]. Furthermore, infarcts or transient ischemic attacks involving these regions significantly increase the risk of recurrent ischemic events, particularly among patients with advanced arterial stenosis [42]. Hemodynamic and biochemical markers also demonstrate prognostic value. In this cohort, systolic blood pressure (SBP) ranged from 62 mmHg to 272 mmHg. The relationship between SBP and stroke outcomes is complex: although elevated SBP at admission has been associated with lower initial severity and improved outcomes [43], pronounced fluctuations within the first 24 h are linked to worse neurological outcomes, reduced recovery, and higher mortality [44]. Conversely, lower SBP levels appear protective, with recurrence rates reported at 7.2% for SBP < 120 mmHg, 8.7% for SBP 130–140 mmHg, and 14.1% for SBP > 150 mmHg [45]. Regarding biochemical markers, patients with NIHSS scores above 16 demonstrated a mean blood urea nitrogen (BUN) level of 22.60, approximately 2.69 units higher than those with lower scores. Abnormal BUN levels, whether elevated or reduced, have been independently associated with increased ischemic stroke risk, suggesting BUN as a potential marker of stroke severity and prognosis [46]. The RadiomicsClinicCTA dataset has shown that fusion models combining clinical and imaging data can effectively improve the accuracy of collateral circulation assessment in stroke patients [47]. In imaging-based classification, VGG11 has demonstrated strong capability in automatically identifying collateral status from CT images [48]. Additionally, Brainomix e-CTA is now widely adopted in many hospitals as an important tool for automated collateral evaluation, significantly enhancing the efficiency of stroke diagnosis and clinical management [49].

Table 5 presents a comparison of results reported by different researchers who applied Random Forest models to stroke-related outcome prediction. Alemseged et al. analyzed 202 patients and reported an AUC of 0.730. Chernykh et al. evaluated a much larger sample of 5225 patients and achieved an AUC of 0.845. In comparison, the present study, although based on a considerably smaller dataset of 91 patients, achieved the highest AUC of 0.94. This comparison illustrates the modeling strategy used in this study. These findings suggest that a well-designed feature set and appropriate preprocessing approaches can allow a smaller dataset to achieve predictive accuracy that matches or exceeds that of studies with significantly larger sample sizes.

A further limitation of this study lies in the use of retrospective, single-center data. Retrospective designs inherently depend on the accuracy, completeness, and consistency of existing medical records, which may lead to missing variables, inconsistent documentation, or unmeasured confounders. Because the data were not collected under a controlled protocol, certain clinically relevant variables may have been unavailable or incompletely recorded, potentially influencing model performance. Another important limitation is the relatively small sample size. With only 92 cases, the dataset may not provide sufficient variability for machine learning models to learn stable and generalizable patterns. Small sample sizes increase the risk of overfitting, reduce the reliability of parameter estimates, and can cause substantial fluctuations in model performance across cross-validation folds. This instability may limit confidence in the robustness of the predictive patterns identified in this study. Relying on a single institution also introduces institutional bias. Differences in clinical workflows, diagnostic criteria, imaging acquisition protocols, patient demographics, and treatment strategies across hospitals may limit the external validity of the findings. As a result, the predictive patterns identified may not fully generalize to broader or more diverse clinical populations. Single-center datasets often reflect local practices and population characteristics, reducing the model’s ability to perform robustly across heterogeneous healthcare environments.

Future work will prioritize expanding the dataset to multi-center cohorts, allowing for cross-institutional validation to evaluate the model’s robustness across diverse clinical environments. Such external validation can help mitigate institutional bias and greatly enhance the model’s generalizability. Future studies should incorporate prospective data collection to ensure standardized variable measurement, minimize missing data, and capture temporal changes in patient conditions. A prospective or longitudinal study design would further enable dynamic modeling that more accurately reflects real-world clinical decision-making processes and patient trajectories.

## 5. Conclusions

In this study, balancing the data with SMOTE improved predictive performance. Random Forest yielded the strongest results, with 83.3% (CI: 79.1–87.6) accuracy and 84.0% (CI: 79.1–88.9) F1-score, followed by XGBoost with 80.2% (CI: 71.5–89.0) accuracy and 81.4% (CI: 73.8–89.0) F1-score; Logistic Regression improved accuracy to 71.3% (CI: 55.1–87.5) and F1-score to 70.8% (CI: 55.4–86.2), whereas SVM declined accuracy to 52.2% (CI: 37.5–67.0) and F1-score to 51.7% (CI: 36.6–66.8). Model-based and groupwise analyses converged on ipsilateral collateral circulation and unilateral–bilateral stroke as key determinants of prognosis (*p* < 0.001 for both). Among routine clinical indicators, systolic blood pressure was associated with greater severity (*p* = 0.034) and was influenced by diastolic blood pressure, while blood urea nitrogen, a proxy for renal function, showed borderline significance (*p* = 0.059). These findings support the interpretation that absent or poor collateralization may reflect inadequate compensatory perfusion that worsens ischemic injury. Prognosis differed across the unilateral–bilateral stroke used here, and finer lesion mapping in future work may further improve clinical interpretability and predictive accuracy. Building on the predictive capabilities demonstrated in this study, future research should explore the integration of these models into Clinical Decision Support Systems (CDSSs). Incorporating machine learning-based risk stratification tools into CDSS platforms could assist clinicians in the early identification of high-risk patients, support individualized treatment planning, and enhance the overall efficiency of stroke care pathways. Furthermore, prospective validation across multi-center and diverse patient populations, the integration of real-time physiological monitoring, and the development of adaptive or hybrid modeling strategies may further strengthen the clinical robustness and translational potential of these approaches. Together, these advancements have the potential to contribute to more proactive, data-driven, and patient-centered stroke management.

## Figures and Tables

**Table 1:** Comparison of Variables Between Groups.

Variables			Variables (91)	NIHSS Score > 16		*p*-Value
				0 (66)	1 (25)	
Personal Information	Sex-Man		52 (57.14)	37 (56.06)	15 (60.00)	0.735
	Sex-Female		39 (42.86)	29 (43.94)	10 (40.00)	
	Smoking		25 (27.47)	18 (27.47)	7 (28.00)	0.829
	Alcohol consumption		17 (18.68)	11 (16.67)	6 (24.00)	0.496
Disease	Diabetes Mellitus (DM)		22 (24.18)	13 (19.70)	9 (36.00)	0.105
	Hypertension (HTN)		49 (53.85)	36 (54.55)	13 (52.00)	0.828
	Mixed Hyperlipidemia		13 (14.29)	10 (15.15)	3 (12.00)	0.962
	Stroke history		16 (17.58)	12 (18.18)	4 (16.00)	0.918
	Atrial fibrillation AF		24 (26.37)	16 (24.24)	8 (32.00)	0.454
Imaging Examination	Ipsilateral MCAS		63 (69.23)	47 (71.21)	16 (64.00)	0.506
	Contralateral MCAS		30 (32.97)	16 (24.24)	14 (56.00)	0.004
	Unilateral–bilateral stroke		90 (98.90)	65 (98.48)	25 (100.00)	0.024
	Ipsilateral collateral circulation on MRA		46 (50.55)	38 (57.58)	8 (32.00)	0.021
Personal information	Age		65.16 (25–88)	64.30 (25–87)	60.77 (31–88)	0.213
	Systolic blood pressure		165.70 (62–272)	161.38 (112–272)	187.56 (62–253)	0.050
	Diastolic blood pressure		87.00 (42–134)	85.52 (48–134)	90.92 (42–133)	0.609
	Total cholesterol		161.35 (11–265)	159.94 (11–241)	162.10 (95–265)	0.505
	HsCRP		5.15 (0.1–31.98)	4.27 (0.1–21)	7.77 (0.3–31.98)	0.239
	BUN		20.43 (6–98)	19.91 (6–98)	22.60 (11–59)	0.074
	Creatinine		0.96 (0.26–3.12)	0.90 (0.26–3.12)	0.94 (0.45–2.57)	0.118
	Uric acid		6.20 (2.5–66)	6.29 (2.5–66)	5.66 (3.6–9.2)	0.106
	FPG		144.24 (67–438)	136.09 (67–321)	153.08 (89–438)	0.077
Imaging Examination	CCA RI	Ipsilateral side	0.81 (0.34–1)	0.80 (0.34–1)	0.84 (0.61–1)	0.192
		Contralateral side	0.81 (0.45–1)	0.80 (0.45–1)	0.85 (0.62–0.99)	0.092
	ICA RI	Ipsilateral side	0.73 (0.51–1.77)	0.70 (0.52–1)	0.74 (0.51–1)	0.010
		Contralateral side	0.71 (0.41–1)	0.70 (0.41–1)	0.72 (0.5–0.89)	0.086
	Plaque Index	Ipsilateral side	2.30 (0–11)	1.89 (0–11)	2.54 (0–11)	0.012
		Contralateral side	2.45 (0–10)	2.15 (0–10)	2.92 (0–8)	0.061

**Table 2:** Group-wise comparison of characteristics before and after SMOTE.

Variable	Non-Smote			Smote		
	0 (*N* = 66)	1 (*N* = 25)	*p*-Value	0 (*N* = 66)	1 (*N* = 66)	*p*-Value
Contralateral MCA stenosis (CTA)	16 (24.24)	14 (56.00)	0.004	16 (24.24)	25 (37.88)	0.132
Unilateral–bilateral stroke	65 (98.48)	25 (100.00)	0.024	65 (98.48)	66 (100.00)	0.000
Ipsilateral collateral flow (MRA)	38 (57.58)	8 (32.00)	0.021	38 (57.58)	12 (18.18)	0.000
SBP	161.38 (112–272)	187.56 (62–253)	0.050	161.38 (112–272)	171.86 (62–263)	0.034
BUN	19.91 (6–98)	22.60 (11–59)	0.074	19.91 (6–98)	20.14 (11–59)	0.059
FPG	136.09 (67–321)	153.08 (89–438)	0.077	136.09 (67–321)	153.73 (67–438)	0.023
Contralateral CCA resistance index	0.80 (0.45–1)	0.85 (0.62–0.99)	0.092	0.80 (0.45–1)	0.85 (0.62–1.0)	0.001
Ipsilateral ICA resistance index	0.70 (0.52–1)	0.74 (0.51–1)	0.010	0.70 (0.52–1)	0.79 (0.51–1.77)	0.007
Contralateral ICA resistance index	0.70 (0.41–1)	0.72 (0.5–0.89)	0.086	0.70 (0.41–1)	0.73 (0.5–0.91)	0.297
Plaque index (ipsilateral)	1.89 (0–11)	2.54 (0–11)	0.012	1.89 (0–11)	2.8 (0–11)	0.002
Plaque index (contralateral)	2.15 (0–10)	2.92 (0–8)	0.061	2.15 (0–10)	2.71 (0–9)	0.066

**Table 3:** Performance of classification models under the imbalanced Non-SMOTE condition.

	AUC	Accuracy	Precision	Recall	F1-Score
Logictic Regression	0.682 (CI: 0.605–0.759)	69.1% (CI: 58.1–80.0%)	49.7% (CI: 12.9–86.4%)	24.0% (CI: 16.2–31.8%)	30.3% (CI: 21.2–39.4%)
Random Forest	0.682 (CI: 0.502–0.862)	69.1% (CI: 58.3–80.0%)	16.7% (CI: −12.6–45.9%)	8.3% (CI: −6.3–23.0%)	11.1% (CI: −8.4–30.6%)
XGBoost	0.575 (CI: 0.412–0.738)	64.8% (CI: 56.7–72.9%)	22.0% (CI: −15.7–59.7%)	16.7% (CI: −12.6–45.9%)	18.9% (CI: −13.9–51.7%)
SVM	0.432 (CI: 0.302–0.561)	72.5% (CI: 65.2–79.7%)	NA	NA	NA

**Table 4:** Performance of classification models after applying the SMOTE.

	AUC	Accuracy	Precision	Recall	F1-Score
Logistic Regression	0.747 (CI: 0.588–0.905)	71.3% (CI: 55.1–87.5%)	71.2% (CI: 53.4–88.9%)	70.7% (CI: 56.9–84.4%)	70.8% (CI: 55.4–86.2%)
Random Forest	0.94 (CI: 0.9–0.98)	83.3% (CI: 79.1–87.6%)	79.6% (CI: 71.6–87.7%)	89.4% (CI: 81.8–97.1%)	84.0% (CI: 79.1–88.9%)
XGBoost	0.907 (CI: 0.847–0.966)	80.2% (CI: 71.5–89.0%)	78.3% (CI: 64.6–92.0%)	86.6% (CI: 73.6–99.7%)	81.4% (CI: 73.8–89.0%)
SVM	0.398 (CI: 0.239–0.558)	52.2% (CI: 37.5–67.0%)	56.3% (CI: 38.2–74.4%)	55.5% (CI: 20.9–90.1%)	51.7% (CI: 36.6–66.8%)

**Table 5:** Comparison of Random Forest AUC Performance Across Different Studies.

	Number of Patients	Selected Model	Performance Comparison
Alemseged et al. [50]	202	Random Forest	AUC: 0.730
Chernykh et al. [51]	5225	Random Forest	AUC: 0.845
Our Method	91	Random Forest	AUC: 0.94

## Data Availability

The data presented in this study are available on request from the corresponding author. The data are not publicly available due to privacy concerns.
